# Availability and Promotion of Healthful Foods in Stores and Restaurants ― Guam, 2015

**DOI:** 10.5888/pcd14.160528

**Published:** 2017-07-13

**Authors:** Elizabeth A. Lundeen, Brenna K. VanFrank, Sandra L. Jackson, Brittani Harmon, Alyssa Uncangco, Patrick Luces, Carrie Dooyema, Sohyun Park

**Affiliations:** 1 Division of Nutrition, Physical Activity, and Obesity, National Center for Chronic Disease Prevention and Health Promotion, Centers for Disease Control and Prevention, Atlanta, Georgia; 2 Division for Heart Disease and Stroke Prevention, National Center for Chronic Disease Prevention and Health Promotion, Centers for Disease Control and Prevention, Atlanta, Georgia; 3 Bureau of Community Health Services, Guam Department of Public Health and Social Services, Mangilao, Guam

## Abstract

Chronic disease, which is linked to unhealthy nutrition environments, is highly prevalent in Guam. The nutrition environment was assessed in 114 stores and 63 restaurants in Guam. Stores had limited availability of some healthier foods such as lean ground meat (7.5%) and 100% whole-wheat bread (11.4%), while fruits (81.0%) and vegetables (94.8%) were more commonly available; 43.7% of restaurants offered a healthy entrée or main dish salad, 4.1% provided calorie information, and 15.7% denoted healthier choices on menus. Improving the nutrition environment could help customers make healthier choices.

## Objective

Guam, a US Affiliated Pacific Island, has a similarly high prevalence of self-reported obesity and chronic disease as the continental United States; 31.6% of adults have obesity, 32.0% have hypertension, and 12.0% have diagnosed diabetes (1,2). The nutrition environment — the availability, pricing, and promotion of foods in stores and restaurants — may contribute to chronic disease (3). The Guam Department of Public Health and Social Services (DPHSS) and its partners outlined chronic disease prevention strategies, including increasing fruit and vegetable intake and decreasing salt consumption (4). To guide these strategies, we conducted assessments of the nutrition environment in stores and restaurants.

## Methods

A sampling frame of 607 stores and 711 restaurants was developed using business listings from the Guam DPHSS, Division of Environmental Health, Supplemental Nutrition Assistance Program, and the telephone directory. A regionally stratified, disproportionate allocation sampling method was used to randomly select 114 stores, classified as large stores (≥2 cash registers, n = 37) and small stores (1 cash register, n = 77), and 63 restaurants (43 sit-down, 20 fast-casual/fast-food). Stores were surveyed if they were open to the public, had a permanent or nonmobile structure, and sold at least 3 of 5 staple foods (milk, bread, eggs, meat or fish, or produce). Three large stores, where many residents shopped, were deliberately sampled. Restaurants were included if they met the first 2 criteria and offered 5 or more breakfast, lunch, or dinner entrées.

The Nutrition Environment Measures Survey (NEMS) is a validated assessment tool for stores (NEMS–S) (5) and restaurants (NEMS–R) (6). Being a US territory, Guam has food options in restaurants and stores that are similar to those in the continental United States. Additionally, NEMS was further adapted for Guam through a literature review on local dietary patterns (7,8), consultation with local dietitians, and field testing of the survey tools. In September 2015, surveyors used NEMS to assess food options through on-site observations and menu reviews. In stores, NEMS defined healthier options as fruits and vegetables, reduced-fat milk, low-calorie beverages, whole grains, lean meats, and reduced-fat condiments or snacks. Because many stores lacked visible prices, a few commonly available staple foods were preselected for pricing. Within-store price comparisons were made between healthier items and their regular counterparts: reduced-fat (≤2%) milk versus whole milk and brown rice versus white rice. Price comparisons were made between large and small stores for 4 healthier items: bananas, cabbage, reduced-fat milk, and brown rice. In restaurants, healthy dishes were defined based on calorie and fat content or menu icons denoting healthy items. Within-restaurant price comparisons were made for the least expensive healthy entrées and sides versus the least expensive less-healthy entrées and sides. We used χ^2^ tests to examine differences in availability and *t* tests for pricing comparisons (significant at *P* < .05). Analyses were weighted to account for survey design.

## Results

Stores had limited availability for several healthier items like 100% whole-wheat bread (11.4%), whole-grain cereal with less than 7 g of sugar per serving (25.4%), lean ground meat with 10% or less fat (7.5%), and 1% fat/skim milk (20.6%) (Table 1). Availability was greater for other items like fruits (81.0%) and vegetables (94.8%), including fresh fruits (65.3%) and fresh vegetables (62.8%). Among stores that sold fresh fruits and vegetables, variety was somewhat limited; 46.9% sold more than 2 varieties of fresh fruits, and 57.9% sold more than 2 varieties of fresh vegetables. For the majority of healthier foods, availability was greater in large stores than in small stores. There was no difference in the average price of reduced-fat milk ($5.94 per 0.5 gallon) versus whole milk ($5.98 per 0.5 gallon), or brown rice ($6.35 per 5 lb) versus white rice ($6.46 per 5 lb). Small stores had significantly higher prices than large stores for bananas ($1.82 vs $1.44 for 1 lb), reduced-fat milk ($6.06 vs $5.76 for 0.5 gallon), and brown rice ($6.78 vs $6.01 for 5 lb). Stores more commonly promoted less-healthy eating (57.1%) than healthy eating (10.7%) through store signage.

**Table 1:** Availability of Healthier Food Options and Promotion of Healthier Choices and Less-Healthy Choices, by Store Size — Guam, 2015

Survey Item	All Stores, %^a^	By Store Size	
		Large Stores,^b^ %^a^	Small Stores,^c^ %^a^
**Availability of Food Item**			
**Grains (n = 110)^d^**			
100% whole-wheat bread	11.4	30.3^e^	1.3^e^
Brown rice	45.3	80.7^e^	26.3^e^
Whole-grain cereal (<7 g sugar per serving)	25.4	57.6^e^	8.2^e^
**Meat (n = 113)^d^**			
Lean ground meat (≤10% fat)	7.5	21.9^e^	0.0^e^
Reduced-fat hot dogs (≤9 g fat per serving)	27.5	47.4^e^	17.2^e^
Fish (frozen or fresh, not breaded)	60.3	91.6^e^	44.0^e^
Canned tuna in water	58.8	82.6^e^	46.4^e^
Reduced-fat Spam (≤8 g fat per serving)	75.3	85.3^e^	70.1^e^
**Fruits and vegetables (n = 114)^d^**			
Any fruit	81.0	93.9^e^	74.1^e^
Fresh fruit	65.3	92.7^e^	50.7^e^
>2 varieties of fresh fruit (n = 71)^d^^,^^f^	46.9	71.6^e^	22.9^e^
Fruit canned in water or 100% juice	61.5	80.7^e^	51.3^e^
Frozen fruit	14.0	40.3^e^	0.0^e^
Any vegetable	94.8	96.5	94.0
Fresh vegetables	62.8	91.7^e^	47.1^e^
>2 varieties of fresh vegetables (n = 70)^d^^,^^f^	57.9	81.0^e^	34.1^e^
Vegetables canned without sauce	94.8	96.5	94.0
Frozen vegetables	71.1	85.7^e^	63.3^e^
**Beverages (n = 108)^d^**			
Low-fat (1%) or skim milk	20.6	49.2^e^	6.0^e^
Diet soda	97.4	100.0	96.1
100% fruit juice	89.7	96.2^e^	86.4^e^
Water	100.0	100.0	100.0
**Condiments or snacks (n = 112)^d^**			
Coconut milk (≤4.5 g fat per serving)	5.6	15.8^e^	0.0^e^
Salad dressing (≤3 g fat per serving)	23.1	40.8^e^	13.4^e^
Baked chips (≤3 g fat per serving)	18.6	26.2	14.4
**Messages and Practices**			
**Healthy promotion and placement practices (n = 112)^g^**			
Promotion of healthy eating through signs or displays^h^	10.7	21.5^e^	4.8^e^
Healthier foods present at the point of purchase	7.6	9.8	6.4
Healthier foods present at the ends of aisles	20.8	27.0	17.5
**Less-healthy promotion and placement practices (n = 112)^g^**			
Promotion of less-healthy eating through signs or displays^i^	57.1	69.9^e^	50.1^e^
Less-healthy foods present at the point of purchase	96.6	96.4	96.7
Less-healthy foods present at the ends of aisles	91.5	91.2	91.6

a Weighted percentage of stores.

b ≥2 Cash registers.

c 1 Cash register.

d Unweighted sample size. For each group of foods, the analysis was limited to stores that had information for all foods in the group. Within any group, some stores were missing data for 1 or more of the foods.

e Indicates significant difference at *P* < .05 between large stores versus small stores.

f Among stores selling fresh fruit or vegetables, variety was defined as different types of fruits or vegetables (eg, apples, bananas, pears).

g Unweighted sample size.

h Signs or displays that promoted healthy eating, including the consumption of fresh or frozen fruits and vegetables, 100% whole-wheat bread, brown rice, whole-grain cereals that are low in sugar, healthy protein such as lean meat (chicken or fish) or beans, grilled chicken or fish rather than fried, fat-free or low-fat dairy products (milk, yogurt, cheese), water or 100% fruit juice, or healthier versions of snack foods (eg, baked chips rather than fried).

i Signs or displays that promoted unhealthy eating, including the consumption of sugar-sweetened beverages, fried foods, foods high in sugar (eg, candy, cookies, sugary cereals), foods high in salt (eg, fried chips), or baked goods high in fat and sugar.

Although only 12.6% of restaurants offered 1 or more healthy entrées on the adult menu, 39.4% had a healthy main salad (Table 2). Combined, 43.7% offered a healthy entrée or main dish salad. Over half (58.8%) offered free refills of sugar-sweetened beverages. Thirty-three percent of restaurants had a kids’ menu; of these, 62.8% offered 1 or more healthy entrées and 48.8% provided a healthy beverage by default. Calorie information was available in 4.1% of restaurants, whereas 15.7% had menu icons denoting healthier dishes. The only significant difference in pricing was found in sit-down restaurants, where the least expensive healthy entrée cost $16.53 on average versus $13.86 for the least expensive less-healthy entrée. Restaurants more commonly used signs and displays to encourage unhealthy eating (29.1%) than healthy eating (19.2%).

**Table 2:** Availability of Healthy and Regular Food Options and Promotion of Healthy and Less-Healthy Choices, by Restaurant Type — Guam, 2015^a^

Survey Item	All Restaurants (n = 63), %	By Restaurant Type	
		Sit-Down (n = 43), %	Fast-Casual/Fast-Food (n = 20), %
**Availability**			
**Entrées and salads on adult menu**			
≥1 healthy entrées^b^	12.6	9.1	20.9
≥1 healthy main salads^b^	39.4	35.5	48.9
≥1 healthy entrées or main salads	43.7	39.9	53.0
≥1 healthier-preparation entrées^c^	92.7	90.1	99.3
**Sides and healthy alternatives on adult menu**			
≥1 healthy fruit side^d^	29.6	37.0	11.4
≥1 healthy vegetable side^e^	61.5	72.5^f^	34.8^f^
Baked chips (n = 14)^g^	9.0	0.0	14.4
100% whole-wheat bread (n = 40)^g^	5.6	8.0	0.0
Brown rice (n = 52)^g^	11.6	11.1	12.9
**Beverages**			
≥1 healthier or low-calorie beverages^h^	100.0	100.0	100.0
≥1 sugar-sweetened beverages^i^	96.9	95.6	100.0
Free refills on sugar-sweetened beverages	58.8	61.2	53.0
Low-fat (≤1%), unflavored milk	9.8	4.5	22.8
**Kids’ menu**			
Kids’ menu available (n = 63)	32.7	39.0	17.5
≥1 healthy entrées (n = 20)^j^^,^^k^	62.8	59.6	80.5
≥1 healthy sides (n = 20)^j^^,^^l^	77.8	77.3	80.5
≥1 healthy desserts (n = 20)^j^^,^^m^	12.0	0.0	76.4
Healthier drinks^h^ are default beverage (n = 18)^n^	48.8	44.9	65.1
Free refills on unhealthy drinks (n = 20)^j^^,^^i^	50.2	56.7	15.4
**Menu labeling**			
Calorie content information	4.1	0.0	14.1
Fat content information	3.9	0.0	13.4
Calorie and fat content information	3.9	0.0	13.4
“Healthy” menu icons or lighter fare section	15.7	9.7	30.2
Fat and calories content information or “healthy” menu icons or lighter fare section	18.8	9.7	40.9
**Messages and Practices**			
**Messages promoting healthy choices^o^**			
Highlight healthy menu options	17.7	11.4	32.9
Encourage healthy eating^p^	19.2	14.7	30.2
Promote free refills on healthier or low-calorie drinks	11.0	9.9	13.4
**Messages promoting less-healthy choices** o			
Encourage unhealthy eating^q^	29.1	16.7^f^	59.1^f^
Encourage overeating	4.7	4.4	5.4
Promote free refills on sugary drinks	14.9	19.9	2.7

a Unweighted sample size and weighted percentage are presented.

b Defined using Nutrition Environment Measures Survey (NEMS) calorie and fat criteria, or healthy icons or lighter fare sections on menu. If calorie and fat information were available, an entrée or main dish salad could be considered healthy if it had ≤800 calories (≤650 calories for burgers or sandwiches), ≤30% of calories from fat, and ≤10% of calories from saturated fat. If calorie and fat information were not available, a salad could still be considered healthy if it had ≤2 high-fat ingredients, and if low-fat (≤3 g of fat/serving) or fat-free dressing (0 g of fat per serving) was available, or if dressing could be ordered on the side.

c Defined as poultry, fish, or main dish vegetables prepared using healthier methods (eg, grilled or baked).

d Fruit with no added sugar, syrup, glaze, or sauce.

e Nonfried vegetables with no added sauce. Side salads counted as a healthy vegetable side if the restaurant had low-fat or fat-free dressing (or dressing could be ordered on the side).

f Significant difference at *P* < .05 between sit-down and fast-casual/fast-food.

g Calculated only for restaurants that served chips, bread, or rice, respectively.

h Healthier beverages: diet soda, water, 100% fruit juice, unsweetened tea or coffee, and unflavored low-fat milk.

i Sugar-sweetened beverages: soda, juice drink, flavored milk, sweetened tea, and energy or sports drinks.

j Calculated only for the 20 restaurants with a kids’ menu.

k Defined by preparation method (ie, grilled or baked rather than fried), “healthy” menu icons or “lighter fare” sections, and other criteria (ie, not prepared with red meat, cheese, or cream sauce).

l Fruit without added sugar, nonfried vegetables without added sauce, rice, salad, beans, low-fat yogurt, cottage cheese, or applesauce.

m Low-fat yogurt or fruit without added sugar.

n This question was only applicable for 18 restaurants that had a default beverage assigned to the kids’ menu.

o Messages on signs, table tents, and displays.

p Messages encourage healthy eating; for example, by choosing fruits and vegetables, reduced fat menu options, baked or grilled foods rather than fried foods, or brown rice instead of white rice.

q Messages encourage unhealthy eating; for example, by choosing desserts that are high in calories or fat, fried foods, or sugar-sweetened beverages.

## Discussion

Availability of healthier foods in stores varied across items, with some healthier options having limited availability. Small stores generally had less availability and higher prices for healthier foods than did large stores. In restaurants, availability of healthier foods was limited and nutrition information was generally unavailable. Promotional materials in stores and restaurants more commonly encouraged unhealthy eating. These findings are similar to the findings of surveys in the continental United States (5,9–11) and American Samoa (12), which found more limited availability of healthier options in small stores. Findings from restaurants in Guam are consistent with findings from a restaurant survey in Minnesota, which found limited availability of healthy entrées and nutrition information (11).

This study is the only nutrition environment assessment of its kind in Guam. By assessing both stores and restaurants, the survey provided a comprehensive picture of the nutrition environment on the island. However, this assessment has limitations. Challenges in creating an accurate sampling frame because of business closures, name changes, and venue duplication may have influenced sampling weights. Store price comparisons were restricted by the absence of displayed prices in most small stores. Additionally, the assessment of healthy entrées in restaurants was limited by the lack of nutrition information or menu icons denoting healthy items in most restaurants.

These findings can be used to develop strategies to help customers choose healthier options. Stores and restaurants could increase the availability of healthy foods and promote the healthy foods they offer. Promotion strategies could include encouraging healthy eating through signage, providing menu icons to denote healthy dishes in restaurants, assigning healthier beverages as the default for kids’ menus, or placing healthier foods in more prominent locations in stores. Improving the availability, pricing, and promotion of healthier options in stores and restaurants could help efforts to increase consumption of healthful foods in Guam.
